# Advances in the Synthesis of Ferrierite Zeolite

**DOI:** 10.3390/molecules25163722

**Published:** 2020-08-14

**Authors:** Hao Xu, Jie Zhu, Longfeng Zhu, Enmu Zhou, Chao Shen

**Affiliations:** 1 College of Biology and Environmental Engineering, Zhejiang Shuren University, Hangzhou 310015, China; xuhao@zju.edu.cn (H.X.); geormochou@outlook.com (E.Z.); 2 College of Biological, Chemical Science and Engineering, Jiaxing University, Jiaxing 314001, China; zhuyaojie1113@163.com

**Keywords:** ferrierite zeolite, synthesis route, organic template, organotemplate-free synthesis, morphology control, mesopore, heteroatom

## Abstract

As one of the most important porous materials, zeolites with intricate micropores have been widely employed as catalysts for decades due to their large pore volume, high surface area, and good thermal and hydrothermal stabilities. Among them, ferrierite (FER) zeolite with a two-dimensional micropore structure is an excellent heterogeneous catalyst for isomerization, carbonylation, cracking, and so on. In the past years, considering the important industrial application of FER zeolite, great efforts have been made to improve the synthesis of FER zeolite and thus decrease the synthesis cost and enhance catalytic performance. In this review, we briefly summarize the advances in the synthesis of FER zeolite including the development of synthesis routes, the use of organic templates, organotemplate-free synthesis, the strategies of morphology control, and the creation of intra-crystalline mesopores. Furthermore, the synthesis of hetero-atomic FER zeolites such as Fe-FER and Ti-FER has been discussed.

## 1. Introduction

Zeolites are crystalline microporous materials which have been widely applied on an industrial scale in heterogeneous catalysis, adsorption, separation and ion exchange in the past decades [1,2,3,4,5]. Among them, Ferrierite (FER) zeolite is one of the industrialized types, which has excellent catalytic properties in skeletal isomerization of n-alkene, methanol to olefin, N_2_O decomposition, CO_2_ hydrogenation, dehydration of methanol or ethanol, dimethyl ether carbonylation, and so on [6,7,8,9,10,11,12,13,14,15,16].

FER is a medium-pore-size zeolite containing two perpendicular intersecting channels. One is 8-membered ring (MR) channel (3.4 × 5.4 Å) along the [010] direction, and the other one is the 10 MR channel (4.3 × 5.5 Å) along the [001] direction [17,18,19]. Since the first synthetic preparation of FER zeolites in the 1960s, many efforts have been made in the synthesis and modification of FER zeolites in the past decades considering the important industrial application of FER zeolite in skeletal isomerization of n-butene. Therefore, FER zeolites with different elementary composition, morphology, hierarchical structure, acid strength, and catalytic properties have been successfully prepared using different synthesis methods [12,19,20,21,22,23,24,25].

In this review, we briefly summarize the synthesis advances of FER zeolite including the development of synthesis routes, the use of organic templates, organotemplate-free synthesis, the strategies of morphology control, the creation of intra-crystalline mesopores and the isomorphous substitution of skeleton Si and Al atoms with heteroatoms.

## 2. The Routes for the Synthesis of Aluminosilicate FER Zeolite

Up to now, many routes for synthesizing aluminosilicate FER zeolite (namely ZSM-35) have been developed, including hydrothermal synthesis, solvothermal synthesis, vapor phase transport (VPT) synthesis, transformation synthesis, solid-state synthesis, microwave-assisted synthesis and topotactic condensation of a layered precursor [12,25,26,27,28,29,30,31]. The distinct features of each synthesis routes have been listed in Table 1.

As with many other important zeolites, the earliest and most common synthesis route for aluminosilicate FER zeolites is hydrothermal synthesis, which is performed at very high temperature (above 300 °C) in the presence of Na^+^-Ca^2+^, Ca^2+^-Sr^2+^-Na^+^ or Na^+^-tetramethylammonium (TMA) composite [25,32]. Then, with the employment of new organic templates such as cetyltrimethylammonium, diethanolamine, pyrrolidine (THP) or ethylenediamine (EDA), the synthesis temperature could be reduced to around 150 °C [20,33,34,35]. Obviously, the synthesis process at a relatively low temperature condition effectively reduces the energy consumption and also improves the production safety.

Instead of the conventional water solvent used in the hydrothermal synthesis process, organic solvents could also be employed in the synthesis of zeolites, which is known as solvothermal synthesis. In order to grow large aluminosilicate FER zeolite single crystals, Kuperman et al. replaced the H_2_O solvent with non-aqueous solvent to control the solubility of the reagent gel particles. This so-called non-aqueous synthesis system included triethylamine (Et_3_N) as solvent, HF as mineralizer and tetrapropylammonium bromide (TPABr) as an organic template. Giant single crystals of aluminosilicate FER zeolite with the size of around 700 μm were successfully obtained [22]. Moreover, Pang et al. performed the synthesis of aluminosilicate FER zeolites in the alcoholic system in 1995. In ethanol solvent, aluminosilicate FER zeolites could be synthesized using a serious of amine including piperidine (Pi), dibutylamine (DBA), ethylenediamine (EDA) and cinchonine as the organic templates [27].

**Figure 1:**
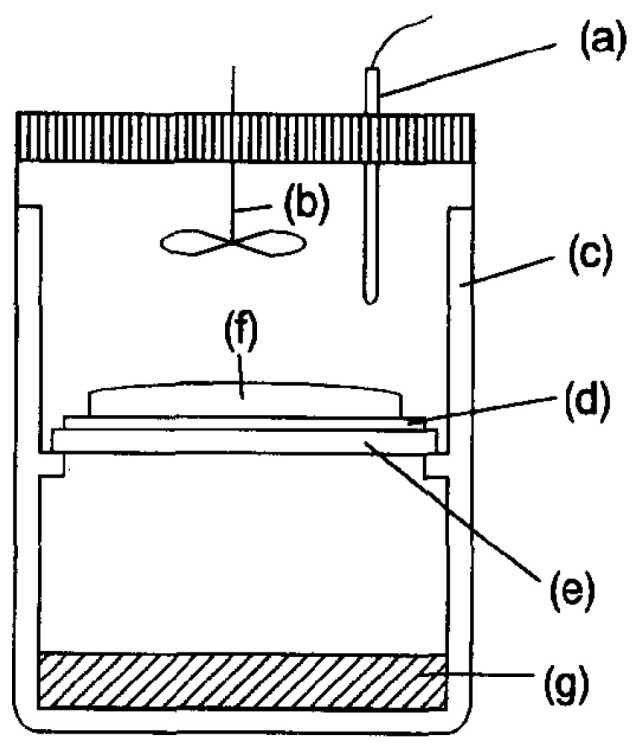
Schematic diagram of special autoclave for the vapor-phase transport synthesis: (**a**) thermocouple, (**b**) agitator, (**c**) Teflon vessel, (**d**) support, (**e**) perforated plate, (**f**) amorphous gel and (**g**) liquid phase. Reprinted with permission from ref. [28]. Copyright 1996, Elsevier.

Later, Matsukata et al. reported a vapor-phase transport (VPT) synthesis route for the preparation of aluminosilicate FER zeolite. In this process, dry aluminosilicate gel was prepared by mixing aging, and drying silica and alumina source. Then, the vapor containing EDA, Et_3_N and H_2_O was supplied to the dry-gel at 180 °C for several days (Figure 1). The roles of water and amines in this synthesis system were investigated in detail, giving the result that EDA acted as OSDA while Et_3_N and H_2_O promoted crystallization. As a result, the dry-gel could be fully converted into zeolite and thus enhanced both the solid yield and the Si/Al ratio (14.5–15.5) of the product [28]. Long et al. reported a novel VPT synthesis of high-silica FER zeolite (Si/Al = 9.8). In this report, they used the mixture of tetrahydrofuran (THF) and water as a vapor phase for the first time. THF in the vapor phase played the template role, while FER zeolite seeds in the dry-gel and H_2_O in the vapor phase promoted the crystallization [36].

Transformation synthesis, which is another synthesis route for synthesizing aluminosilicate FER zeolite, was performed in 1998 by Beyer et al. In this work, high-silica FER zeolite (Si/Al = 15–18) could be synthesized by recrystallization of magadiite in the dry state (H_2_O/SiO_2_ = 8.7 in the starting mixture) and aqueous suspension (H_2_O/SiO_2_ = 40 in the starting mixture) with the piperidine as an organic template. It is worth noting that the crystallization time of aluminosilicate FER zeolite in dry state system was much shorter than that in aqueous suspension. Moreover, the crystal morphology of the two products was different. However, the reasons for the above phenomena were not clarified [29].

From the viewpoint of green chemistry, the use of a solvent in the above synthesis of aluminosilicate FER zeolites would produce a large amount of polluted water, which is not a sustainable manner [42,43,44,45]. Encouragingly, the solvent content in the crystallization of aluminosilicate FER zeolites was significantly reduced using VPT route and transformation synthesis route [28,29,36]. However, in the VPT synthesis route, the preparation of dry-gel still needs a large amount of water as a solvent [28,36]. It is worth noting that the H_2_O/SiO_2_ in the above-mentioned dry state transformation synthesis system is still not low enough (8.7) [29]. In 2000, Beyer et al. reported a novel synthesis route for high-silica FER zeolite (Si/Al = 12–35) by solid-state recrystallization of aluminum-containing kanemite varieties using piperidine as an organic template. This work was progressive that water solvent was not necessary in the crystallization of FER zeolite. However, similar to the VPT route, the preparation of kanemite could not avoid the use of a large amount of water solvent [37]. Moreover, Dou et al. reported a solid-transformation synthesis route of aluminosilicate FER zeolite using conventional silica and aluminum source (fumed silica and aluminum sulfate). In this synthesis route, although the H_2_O/SiO_2_ ratio was successfully reduced to 2.6, the amount of ethylene diamine (EDA) as an organic template added in the synthesis system was relatively high (EDA/SiO_2_ = 0.95) [30]. 

In addition, since the first successful application of microwave-assisted synthesis of ZSM-5 zeolite reported by Mobil researchers in 1988, this synthesis method has attracted a lot of attention because of its unique advantages such as fast crystallization, high efficiency, and simple preparation [46,47,48,49]. Recently, Xu et al. reported a rapid synthesis of aluminosilicate FER zeolites through microwave-assisted route. Comparing to the conventional crystallization time (several days), the crystallization rate was greatly enhanced in this preparation and the aluminosilicate FER zeolite could be obtained within only 2–3 h in the absence of OSDA [31].

Furthermore, considering the bottom-up synthesis concept, FER zeolite could also be obtained by topotactic conversion from its layered precursors. The first layered precursor named as PREFER was synthesized by Marler et al. in fluoride media with the use of a bulky template, 4-amino-2,2,6,6-tetramethylpiperidine. After calcination at around 500 °C, the PREFER layers would link together through condensation reactions and thus transform to 3-dimentional (3D) FER topology [38]. Since then, many other layered precursors of FER zeolite, such as PLS-3 and ICP-2 were gradually obtained by the different synthesis routes [39,40,41].

## 3. Synthesis of Aluminosilicate FER Zeolite with the Use of the Different Organic Templates

In the zeolite synthesis, the application of organic templates has many advantages such as reducing the crystallization temperature and time, increasing the Si/Al ratio of the products, adjusting the properties of the products and so on. So far, a variety of organic templates, including alkylamines, cyclic amines, and alcohols have been reported to be used in the synthesis of aluminosilicate FER zeolites [6,18,20,26,27,29,32,36,37,50,51,52,53,54,55,56,57,58,59,60,61,62,63,64,65,66,67,68,69]. The detailed information of organic templates for synthesizing FER zeolite is shown in Table 2.

Since the 1970s, tetramethylammonium (TMA), ethylenediamine (EDA), and pyrrolidine (THP) were the earliest organic templates used in the synthesis of aluminosilicate FER zeolite [20,32]. For instance, Mobil researchers reported the hydrothermal synthesis of aluminosilicate FER zeolite with the use of EDA or THP [20]. 

Except for the TMA and EDA as the organic templates for synthesizing FER zeolites, some other alkylamines could also be used as the organic templates in the preparation of aluminosilicate FER zeolites. Examples include the solvothermal synthesis using dibutylamine (DBA) [27], the hydrothermal synthesis with the use of *n*-butylamine (*n*-BA) [54], 1,8-diaminooctane (DAO) [55], and isopropylamine (IPA) [56].

Other than the THP as an organic template for synthesizing FER zeolites, some other cyclic molecules, which have the similar molecular structure to THP, such as pyridine (Py) [57,58], piperidine (Pi) [6,29,37], tetrahydrofuran (THF) [26,36,60], *N*,*N*-diethyl-*cis*-2,6-dimethyl piperidinium (DMPi) [18,61] and 1,3-dimethyimidazolium (DMI) [62,63] could also be used as the organic templates in the synthesis of aluminosilicate FER zeolites. For example, in 1989, Smith et al. reported the hydrothermal synthesis of aluminosilicate FER zeolite with the range of Si/Al ratios at 15.1–19.3 using Py as an organic template [57]. Later, in 1999, Martínez et al. successfully synthesized high silica FER zeolite (Si/Al = 59) with the use of Py as an organic template under rotation condition. The obtained high silica FER zeolite exhibited high selectivity as well as high stability during the skeletal isomerization reaction of *n*-butenes [58]. Moreover, in 1997, a mixture of MOR and FER zeolites were synthesized with the use of Pi as an organic template for the first time [59]. Later, Beyer et al. obtained a pure phase of aluminosilicate FER zeolite by solid-state recrystallization of magadiite or kanemite using Pi as an organic template [29,37]. Recently, a pure phase of aluminosilicate FER zeolite with high crystallinity could be directly synthesized with the use of Pi as an organic template by carefully adjusting the ratio of the starting gel. The obtained product exhibited good catalytic performance in the skeletal isomerization reaction of 1-butene [6]. Meanwhile, Long’s group reported the successful synthesis of FER zeolite with the use of THF as an organic template under the hydrothermal and VPT condition [36]. More recently, we proved that the DMPi molecules could also be used as an organic template for the synthesis of aluminosilicate FER zeolite and thus obtained the nanosheet-like FER zeolite with the thickness at 6–8 nm [61]. 

Moreover, aluminosilicate FER zeolites could be successfully synthesized using the alcohol molecules as the organic templates as well. For examples, Candamano et al. reported the hydrothermal synthesis of (Fe, Al) FER zeolite with the use of ethylene glycol (EG) [64]. Meng et al. performed the hydrothermal synthesis of aluminosilicate FER zeolite in the presence of a large amount of EG (EG/SiO_2_ = 7) [65].

**Figure 2:**
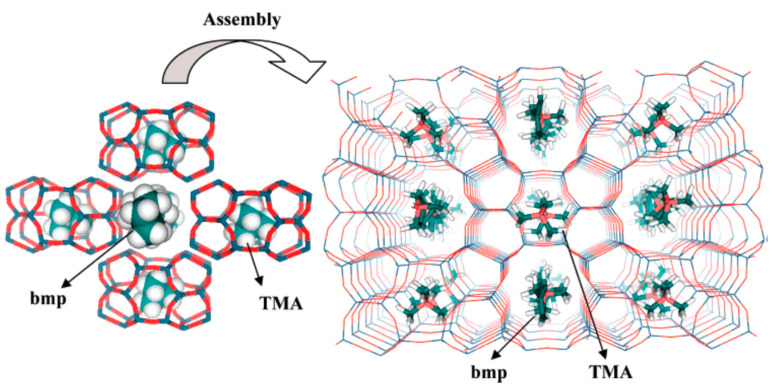
Schematic representation of the self-assembly of tetramethylammonium (TMA)-filled cavities around benzylmethylpyrrolidinium (BMP) molecules to give the final FER structure. Reprinted with permission from ref. [66]. Copyright 2007, American Chemical Society.

In general, it is well-known that the use of different organic templates would affect the properties of the obtained zeolites including Al distribution, acidic strength, morphology and so on, and thus further influence its catalytic performance. In this case, the use of cooperative structure-directing agents (co-SDAs) would attract much attention because the different combinations of the organic templates used in the synthesis could influence the zeolite properties [22,66,70,71,72,73,74]. For example, Perez-Pariente et al. designed a novel synthesis strategy in fluoride medium based on the combination of the small molecules (TMA) with the bulkier molecules (benzylmethylpyrrolidinium, BMP) as the organic templates. Molecular mechanics calculations revealed that TMA molecules sat in the FER cages, while BMP molecules positioned in the 10-MR channels (Figure 2). Following this synthesis strategy, aluminosilicate FER zeolites with the Si/Al ratio in the range of 10–16 were successfully prepared, although the crystallization time was relatively long (at least 10 days) [66]. In 2011, Komarneni et al. prepared the large single crystals of aluminosilicate FER zeolite using the mixed organic molecules (triethylamine (Et_3_N), TPABr, Py, and propylamine (PA)), while the structure-directing effect of these organic molecules was not mentioned [67]. Meanwhile, Davis et al. reported the synthesis of aluminosilicate FER zeolites using a combination of organic templates including TMA and one of the cyclic amines (THP, hexamethyleneimine (HMI), and 1,4-diazabicyclo [2.2.2] octane (DAB)). In this synthesis process, the structure-function relations between the organic template molecules and aluminum distribution within the FER products were carefully studied, giving a result that the location of acidic sites could be adjusted by the use of different organic templates [68]. Later, Wang et al. also used a combination of TMA and a cyclic amine (Pi) as the cooperative organic templates, resulting in the hierarchical aluminosilicate FER zeolite aggregated by nanosheets morphology [75]. Almeida et al. combined a bulky dicationic ion, 1,6-bis(*N*-methylpyrrolidinium) hexane (MPH), and a smaller species, TMA as co-SDAs to control the Al distribution in the synthesis of aluminosilicate FER zeolites [69]. Recently, Rima et al. studied the synthesis of FER zeolites through the combined use of TMA and THP as co-SDAs. The result showed that THP plays an important role in the initial nucleation while TMA^+^ provided both space-filling and basicity capacities in the synthesis of aluminosilicate FER zeolite [71].

## 4. Organotemplate-Free Synthesis of Aluminosilicate FER Zeolite

Even though the aforementioned works are inspiring, considering the industrial production cost and environmental protection, there is no doubt that the idea of complete avoidance of organic templates in the synthesis is particularly attractive [43,76,77,78,79].

The first example for the organic template-free synthesis of aluminosilicate FER zeolite was achieved by Weitkamp et al. with the addition of FER zeolite seeds. The obtained products possessed good crystallinity, typical blocky morphology and a large number of accessible acid sites. However, the range of Si/Al ratio of the products was narrow (6.6–7.8) [80]. Later, Okubo et al. further optimized the synthesis condition of aluminosilicate FER zeolite without the use of organic template or zeolite seeds. In addition, the cooperative effect of sodium and potassium ions was studied in detail. However, the Si/Al ratio of the obtained products was not mentioned in the article. Considering the Si/Al ratio in the starting gel no more than 10, it is reasonable to assume that the Si/Al ratio of the products would not be higher than 10 [81]. Okubo et al. also mentioned the synthesis of aluminosilicate FER zeolite using itself as seeds without the help of an organic template. In this report, the FER product showed a relatively low Si/Al ratio (7.6) and solid yield (22%) [82].

**Figure 3:**
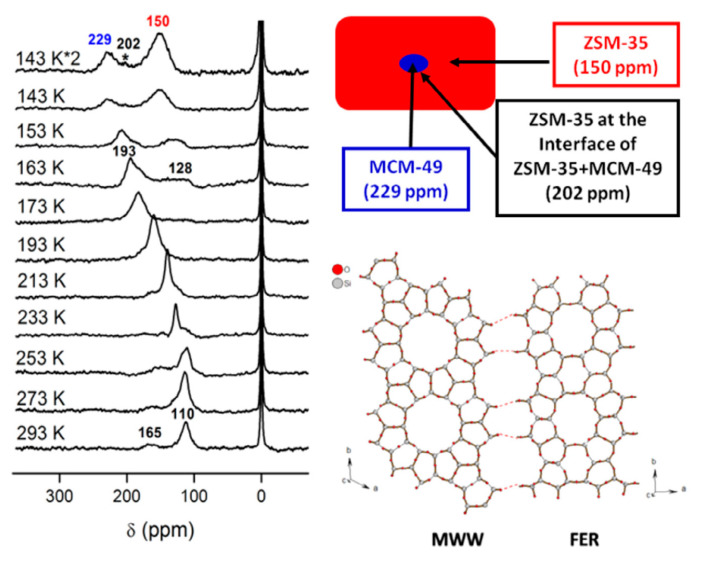
Temperature-dependent hyperpolarized ^129^Xe Nuclear Magnetic Resonance (NMR) spectra of Xe adsorbed in the obtained FER zeolite sample and the possible linkage between the structures of MCM-49 and FER. Reprinted with permission from ref. [87]. Copyright 2014, Elsevier.

Despite the great progress for the organic template-free synthesis of aluminosilicate FER zeolite, the obtained product is always Al-rich (Si/Al ratio no more than 10). Normally, high-silica aluminosilicate FER zeolites could be achieved with the assistance of organic templates [83,84,85,86]. In recent years, it was found that aluminosilicate FER zeolites with relatively high Si/Al ratio could be synthesized by adding zeolite seeds with different topologies from FER, which is regarded as a breakthrough for the synthesis of high silica FER zeolite [21,87]. For examples, Xiao et al. reported the first successful synthesis of high-silica aluminosilicate FER zeolite (Si/Al ratio as high as 14.5, designated as ZJM-2) by inducing CDO-structure zeolite building units in the synthesis system in the absence of any organic template. It is well-known that FER and CDO zeolites have the same building units, and UV-Raman spectra demonstrated the possibility that CDO crystals dissolved into smaller building units under hydrothermal treatment. Therefore, by carefully adjusting the chemical composition in the starting gel and crystallization conditions, aluminosilicate FER zeolite with high silica feature was successfully obtained [21]. Later, Liu et al. reported the organotemplate-free and seed-assisted synthesis of high silica aluminosilicate FER (Si/Al = 14.5) with MCM-49 seeds without the common composite building units with FER structure. Furthermore, the solid yield in this synthesis is relatively high (65–85%). The product exhibits good catalytic performance in DME carbonylation to MA reaction. In addition, a possible growth mechanism is proposed based on the hyperpolarized ^129^Xe Nuclear Magnetic Resonance (NMR) technique (as shown in Figure 3). In this hypothesis, MCM-49 was first dissolved into small fragments in the alkaline gel, then FER framework started to form over MCM-49 through intergrowth connection on the active growth surface (possibly similar 5MR between the two zeolites). This work suggested a new seed-assisted synthesis strategy using the similarity between the seeds and target zeolite [87].

## 5. Morphology Control Strategies of Aluminosilicate FER Zeolite

As mentioned above, aluminosilicate FER zeolite shows excellent catalytic performances in many reactions because of the abundant acidic sites within its uniform micropores. However, the relatively small (below 1 nm) and sole micropores in aluminosilicate FER zeolite often cause diffusion limitations and thus strongly influence the mass transfer in catalysis. Therefore, the synthesis of aluminosilicate FER zeolite nanocrystals is one of the numerous efforts that have been made to overcome the above drawbacks. The access of smaller crystals not only shortens the diffusion paths for reagent and product molecules, but also enlarges the external surface area and thus provides more accessible acidic sites [73,88,89,90].

**Figure 4:**
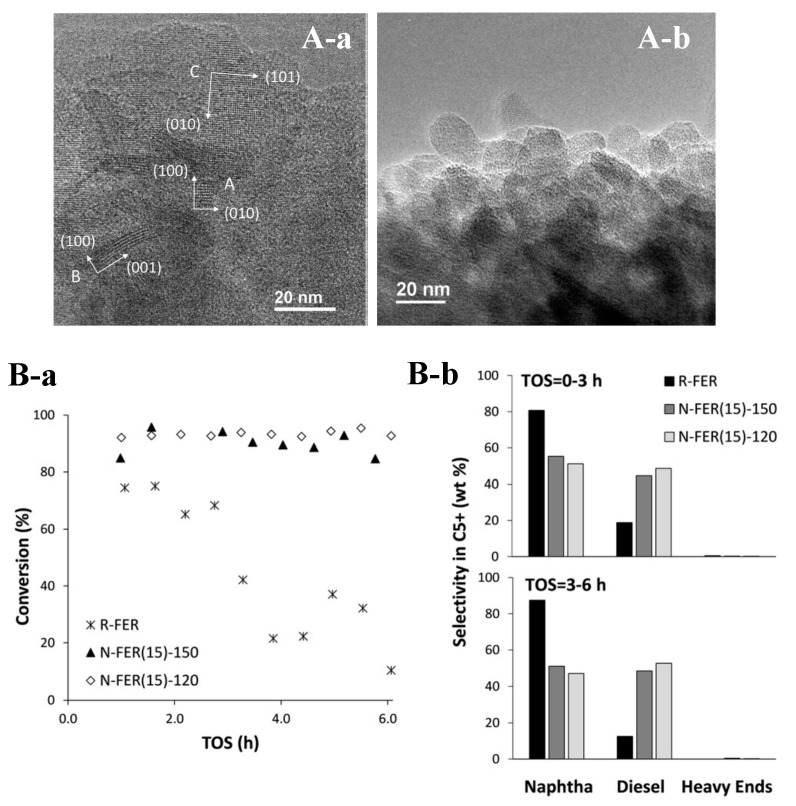
(**A**) TEM images of aluminosilicate FER zeolite nanocrystals synthesized with Si/Al molar ratios of 15 at (**a**) 150 °C and (**b**) 120 °C, respectively. (**B**) Catalytic performance including (**a**) 1-pentene conversion versus time on stream (TOS) and (**b**) selectivity within the C_5+_ liquid fraction in oligomerization of 1-pentene to liquid fuels reactions over aluminosilicate FER zeolite: R-FER (reference FER zeolite synthesized using Pi as a single OSDA), N-FER(15)-150 and N-FER(15)-120 (FER zeolite nanocrystals synthesized with Si/Al molar ratios of 15 at 150 °C and 120 °C). Reprinted with permission from ref. [7]. Copyright 2018, Wiley.

The most common strategy for preparing zeolite nanocrystals is introducing organic surfactants [91,92]. In order to synthesize aluminosilicate FER nanocrystals, Corma et al. used Pi and a modified surfactant (cetylmethylpiperidinium bromide) as the organic templates. The long carbon chain in the surfactant limited the crystal growth of FER zeolites along three dimensions, and thus produced the aluminosilicate FER nanocrystals with sizes in the range of 10–20 nm (Figure 4A). Because of the reduction of crystal size along the [001] direction, the access to Brønsted acid sites increased, which enhanced its catalytic activity as well as lifetime in oligomerization of 1-pentene to liquid fuels reaction (Figure 4B) [7]. Very similarly, Xu et al. reported a successful size control strategy of aluminosilicate FER zeolite by adding cetyltrimethyl ammonium bromide (CTAB) into the synthesis system containing Pi as the organic template. In their report, the crystal size could be adjusted from 100 nm to 2 μm [17]. Later, Hensen et al. reported the transformation synthesis of hierarchically porous aluminosilicate FER zeolite consisted of the agglomerated sheets (~9–15 nm thickness) by using the *N*-methylpyrrolidine and C_16_H_33_-[1,2-dimethyl-3-imidazolium] bromide as the organic templates [12]. Moreover, Limtrakul et al. reported the synthesis of hierarchical aluminosilicate FER nanosheet assemblies with a ball-shaped morphology using the THP and 3-(trimethoxysilyl) propyl octadecyl dimethyl ammonium chloride (TPOAC) as the organic templates [93].

**Figure 5:**
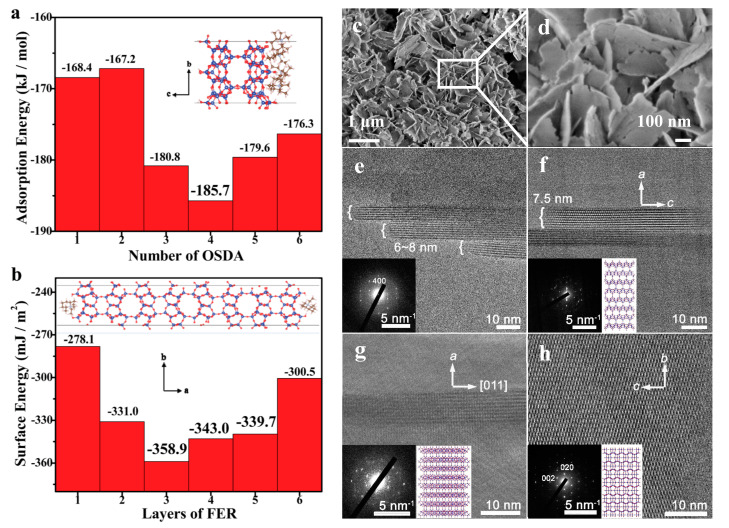
(**a**) Dependence of adsorption energy on FER {100} surfaces on the number of DMPi molecules and its schematic diagram, (**b**) dependence of surface energy on FER {100} on the number of layers of the FER structure and its schematic diagram, (**c**,**d**) SEM images, (**e**–**h**) TEM images of the nanosheets of aluminosilicate FER zeolites and corresponding projections of the FER framework along (**f**) [010], (**g**) [0–11] and (**h**) [100], respectively. Reprinted with permission from ref. [61]. Copyright 2019, The Royal Society of Chemistry.

Notably, the use of surfactants in the synthesis is costly and would cause inconvenience in the product washing process. Thus, it is highly desirable to design a simple and inexpensive synthesis strategy for the nanosized aluminosilicate FER zeolite using the sole and low-cost small OSDAs. Hong et al. reported a synthesis method for aluminosilicate FER nanoneedles with the use of choline as a sole organic template. However, the morphology control mechanism of this synthesis was not mentioned [94]. Recently, we designed a sole small organic ammonium molecule (*N*,*N*-diethyl-*cis*-2,6-dimethyl piperidinium, DMPi) as both structure-directing agent and crystal growth inhibitor (Figure 5a,b). A theoretical calculation showed that when the concentration of DMPi is relatively high, the DMPi molecules would gather on the {100} surface to reduce the adsorption energy, and thus inhibit the growth of FER framework along the [100] direction. With the employment of DMPi, aluminosilicate FER nanosheets with uniform thickness (6–8 nm) could be successfully synthesized (Figure 5c–h). Furthermore, the thickness of the aluminosilicate FER nanosheets could be easily controlled from 6 to 200 nm by just adjusting the DMP concentration in the starting gel. Very importantly, the above-mentioned aluminosilicate FER nanosheets were proven to have the excellent catalytic performance in skeletal isomerization of *n*-butene to isobutene, which might be potentially important for industrial application of this ultrathin nanosheet FER zeolite as a catalyst candidate in the near future [18,61].

Another morphology control strategy of aluminosilicate FER zeolite without the help of organic template was presented by Xu et al. using microwave-assisted synthesis process. In this report, the crystal size of aluminosilicate FER zeolites could be controlled in the range of 0.4–3.0 μm by just adjusting the particle size of the seeds (0.1–1.3 μm). At the same time, the morphology of the products was similar to that of the seed zeolites [31]. This synthesis strategy is very efficient and environmentally friendly. However, high pressure would be produced in the closed vessels under hydrothermal and microwave-assisted condition, which should be especially careful.

On the other hand, large crystals of zeolites also attract a lot of interest because of their unique applications in refining structures and elucidating the intrinsic adsorption and diffusion properties. For instance, Weitkamp et al. reported the solvothermal synthesis of large-crystal all-silica, aluminosilicate, and borosilicate FER zeolites (100–600 μm) in fluoride medium using Py as the solvent. The morphology and crystal size of FER zeolites were monitored by the use of the different alkylamines as the organic templates and chemical composition of the starting gels [95]. Komarneni et al. reported the synthesis of aluminosilicate FER large single crystals by hydrothermal method using the mixed organic structure-directing agents (Et_3_N, THP, PA and TPABr). Notably, the average size of the FER zeolite single crystals would reach to 280 μm, which was very suitable for crystallographic research. However, in this synthesis, ZSM-5 phase could always be found together with FER zeolite, which deserves further investigation [67].

## 6. Creation of Intracrystalline Mesopores in Aluminosilicate FER Zeolite

Another strategy to overcome the diffusion limitation problem of zeolite catalysts is to create intracrystalline mesopores. The introduction of mesopores (2–50 nm) is beneficial to the mass transfer and thus improves the ability of catalytic conversion of bulky molecules [96,97,98].

Creation of the disordered mesopores in zeolite crystals by post-treatments such as steaming and chemical etching has been reported for a long time [99,100]. As for aluminosilicate FER zeolites, Valtchev et al. mixed aluminosilicate FER crystals with HF-NH_4_F solutions at the different HF concentration, which led to the formation of mesopores and macropores. After such treatment, the obtained hierarchical aluminosilicate FER zeolite had similar chemical composition and acid properties to its original one [50]. Recently, Catizzone et al. reported the post-synthesis of hierarchical aluminosilicate FER zeolite by sequential treatments with NaAlO_2_, HCl, and NaOH solutions. The obtained materials exhibit high mesoporous volume, and no significant change on acidity. More importantly, the hierarchical aluminosilicate FER zeolite displayed superiority on methanol conversion in methanol dehydration to dimethyl ether reaction, despite of the formation of by-products at high temperatures [101].

**Figure 6:**
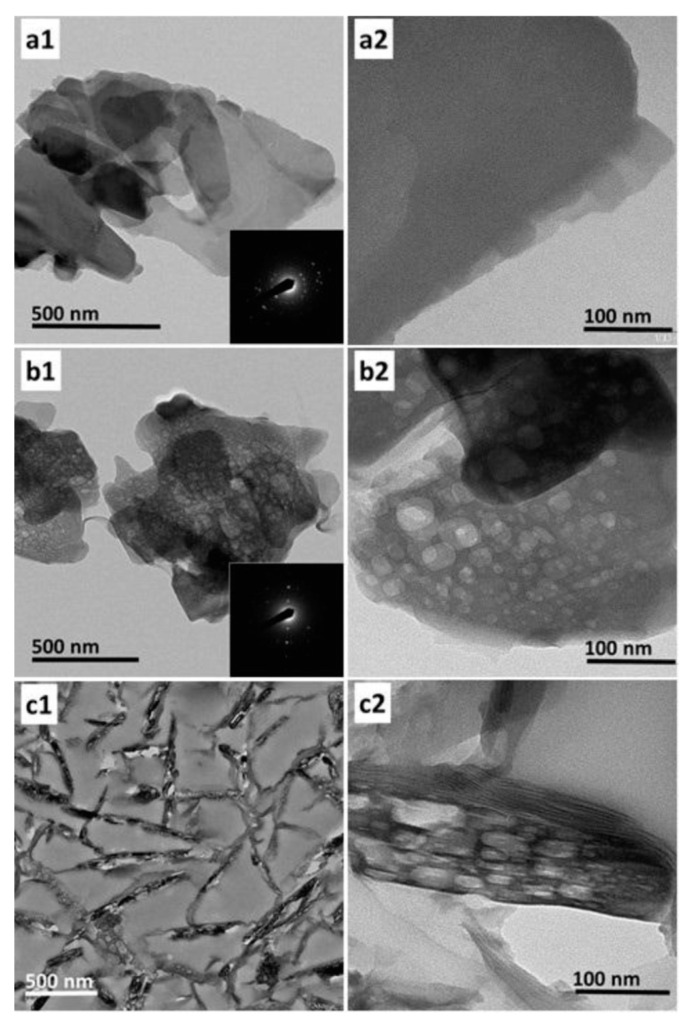
(**a1**–**c1**) low-magnification and (**a2**–**c2**) high-magnification TEM images of FER zeolite samples synthesized with CTAB in NaOH solution with different NaOH concentration of (**a**) 0.05, (**b**) 0.25, and (**c**) 0.5 mol/L at 130 °C for 72 h, respectively. Reprinted with permission from ref. [102]. Copyright 2018, Elsevier.

In addition, it is widely accepted that organic surfactants could also be used to create intracrystalline mesopores in zeolites. Typically, Cheng et al. and Khitev et al. both reported the recrystallization of FER zeolite in alkaline solution in the presence of CATB (Figure 6). In these processes, parent aluminosilicate FER was partially dissolved in alkaline medium. Then, the FER fragments reassembled and thus the CTAB molecules insert between the layers. The mesopores could be successfully formed using this method [102,103].

Compared with the above-mentioned recrystallization process, direct synthesis of zeolites with intracrystalline mesopores is more convenient and cost-effective. To date, direct synthesis of mesoporous zeolites with many topologies such as EUO, BEA, AEI, and CHA were successfully achieved [97,104,105,106]. However, despite much progress in the synthesis of hierarchical aluminosilicate FER with intercrystal mesopores formed by crystal aggregation, there is no report for the direct synthesis of aluminosilicate FER zeolite with intracrystalline mesopores, which needs further investigation.

## 7. Synthesis of Heteroatomic FER Zeolite

In the catalytic application of zeolites, it is desirable to modify the acidity of the zeolite in order to enhance the performance of the zeolite catalysts. Isomorphous substitution of skeleton Si and Al atoms by other elements such as Ge, B, Ga, Fe, Ti and V is one of many good methods to achieve this goal. In that case, heteroatomic FER zeolites with the different elementary composition should be prepared [8,19,107,108,109,110,111,112,113]. The heteroatom contents of heteroatomic FER zeolites are listed in Table 3.

**Figure 7:**
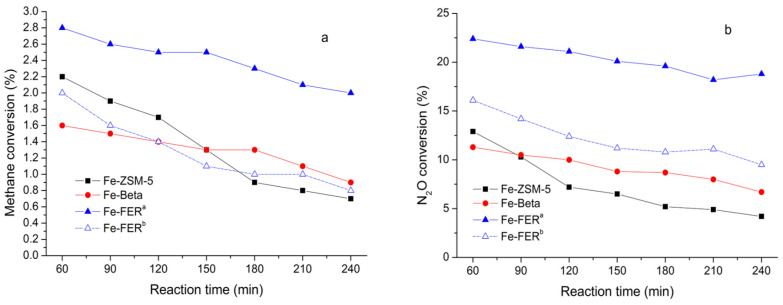
(**a**) Methane conversion and (**b**) N_2_O conversion over the different catalysts at 350 °C (Fe-ZSM-5, Fe-Beta, Fe-FER^a^, 200 mg of catalysts; Fe-FER^b^, 100 mg of catalyst). Reprinted with permission from ref. [110]. Copyright 2019, American Chemical Society.

In the late 1980s, an aluminium-free FER zeolite was synthesized hydrothermally in the presence of EDA and boric acid by Gunawardane et al. [19]. The researchers refer to boric acid as a template, and claim the substitution of Si by B in T-positions might occur at that time, which is now widely recognized as a fact. Later, Ga^3+^ had been successfully incorporated in FER zeolite using the THP as an organic template, resulting in a gallosilicate with FER structure (Si, Ga)-FER [107]. After that, the first synthesis of Fe-FER was also hydrothermally synthesized with the use of THP [108]. Moreover, Kotasthane et al. prepared Fe-FER zeolites with the different Fe/Al ratios and Si/Al ratios. Very interestingly, the Fe-FER samples were found to be active in n-hexane oxidation reactions. Meanwhile, the impacts of synthesis routes and chemical composition of Fe-FER catalysts on the catalytic performance were briefly investigated [109]. Recently, because of the severe NO_x_ pollution, Fe-substituted zeolites were employed as the efficient catalysts in the direct N_2_O decomposition reaction. Among all the Fe-substituted zeolites, Fe-FER was referred to as the best direct N_2_O decomposition catalyst [8]. Furthermore, Stockenhuber et al. compared the activity and selectivity for partial oxidation of methane using N_2_O as oxidant over Fe-ZSM-5, Fe-Beta, and Fe-FER catalysts with similar iron concentrations. The catalytic activity studies showed that Fe-FER was the most active catalyst based on methane and N_2_O conversion (Figure 7) [110]. However, the concentration of framework Al atoms in the zeolite catalysts was different, which led to the different concentration of acid sites in the catalysts and thus influence the catalytic results.

In 1998, Kotasthane et al. prepared a new family of titanium silicate FER (Ti-FER) zeolite using solvothermal synthesis method in the presence of silica-FER seeds [111]. Very similarly, the first successful synthesis of vanadium silicates with FER structure (V-FER) was obtained using a similar solvothermal synthesis route. Electron Paramagnetic Resonance (EPR) spectroscopy confirmed that some of the V^4+^ was in distorted octahedral positions, while Ultraviolet and visible spectrophotometry (UV-vis) and NMR spectroscopy confirmed the presence of tetrahedral V^5+^ species in the frameworks. Moreover, the V-FER exhibited good catalytic properties in toluene oxidation reaction [112].

In addition, another novel synthesis of Ti-FER zeolite was performed by Corma et al. In this report, the titanium-containing laminar analogue TiPREFER was directly synthesized with the use of 4-amino-2,2,6,6-tetramethylpiperidine and HF. After calcination of TiPREFER, the Ti-FER was successfully obtained [113]. However, the obtained Ti-FER presented the low catalytic activity and epoxide selectivity in hex-1-ene epoxidation, which should be further investigated.

## 8. Summary and Future Prospects

In this review, the synthesis of FER zeolite with different methods is discussed in detail. The synthesis routes such as hydrothermal synthesis, solvothermal synthesis, vapor-phase transport, transformation synthesis, solid-transformation synthesis, microwave-assisted synthesis and topotactic conversion are summarized. In addition, the use of different organic templates including alkylamines, cyclic amines, alcohol molecules, and cooperative structure-directing agents are listed separately. Moreover, the development of environmentally friendly organotemplate-free synthesis routes is discussed. Furthermore, the comparisons of different morphology control strategies such as introducing organic surfactants, designing organic template molecules and adjusting the particle size of the zeolite seeds are made. The creation of intracrystalline mesopores through post-treatment or recrystallization with organic surfactant is introduced. Finally, the isomorphous substitution of skeleton atoms of FER zeolite with B, Ga, Fe, Ti, or V atoms is presented.

Despite that, much progress has been made in the synthesis and application of FER zeolites these years, more sustainable synthesis routes, such as the combined strategy of both organotemplate-free and solvent-free routes, and the combined strategy of microwave-assisted synthesis with solvent-free synthesis could be considered. Moreover, the sustainable and effective morphology control strategy and mesopore introduction strategy should be developed. More efforts for preparation of FER zeolite in both fundamental research and industrial production should be made in the future.

## Figures and Tables

**Table 1:** Distinguishable features of synthesis routes for the preparation of aluminosilicate ferrierite (FER) zeolite.

Entry	Synthesis Routes	Features	Ref.
1	Hydrothermal synthesis	water as a solvent	[20,32,33,34,35]
2	Solvothermal synthesis	organic molecules as the solvents	[22,27]
3	Vapor-phase transport (VPT)	vapor containing a small amount of OSDA and water in the dry aluminosilicate gel	[28,36]
4	Transformation synthesis	recrystallization of zeolites with different topologies	[29,37]
5	Solid-transformation synthesis	low water content and relatively high OSDA content	[30]
6	Microwave-assisted synthesis	rapid synthesis with microwave as the energy source	[31]
7	Topotactic conversion	condensation of layered precursors	[38,39,40,41]

**Table 2:** Overview of the reported organic templates, their molecular structures and the corresponding synthesis methods.

Entry	Organic Templates	Structures	Synthesis Method ^1^	Ref
1	tetramethylammonium (TMA)	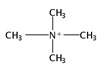	HT	[32]
2	ethylenediamine (EDA)	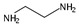	HT, VPT, SS	[30,34,50]
3	pyrrolidine (THP)	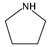	HT	[35,50,51,52,53]
4	dibutylamine (DBA)	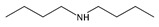	ST	[27]
5	n-butylamine (n-BA)	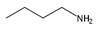	HT	[54]
6	1,8-diaminooctane (DAO)	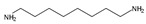	HT	[55]
7	isopropylamine (IPA)	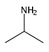	HT	[56]
8	pyridine (Py)	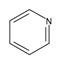	HT	[57,58]
9	piperidine (Pi)	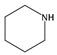	HT, SS,	[6,29,37,59]
10	tetrahydrofuran (THF)	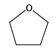	HT, VPT	[26,36,60]
11	*N*, *N*-diethyl-*cis*-2,6-dimethyl piperidinium (DMPi)	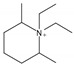	HT	[18,61]
12	1,3-dimethyimidazolium (DMI)	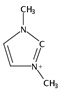	SS	[62,63]
13	ethylene glycol (EG)	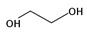	HT	[64,65]
14	benzylmethylpyrrolidinium (BMP)	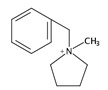	HT (combined with TMA)	[66]
15	hexamethyleneimine (HMI)	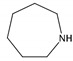	HT (combined with TMA)	[68]
16	1,4-diazabicyclo [2.2.2] octane (DAB)	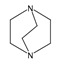	HT (combined with TMA)	[68]
17	1,6-bis (*N*-methylpyrrolidinium)hexane (MPH)	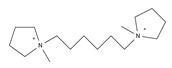	HT (combined with TMA)	[69]

^1^ HT: hydrothermal synthesis, SS: solid-state synthesis, VPT: vapor-phase transport synthesis, ST: solvothermal synthesis.

**Table 3:** Detailed information of heteroatomic FER zeolites.

Entry	Heteroatomic FER Zeolites	Si/Heteroatom Ratio	Ref.
1	B-FER	Not mentioned	[19]
2	Si, Ga-FER	Si/Ga = 6.6	[107]
3	Fe-FER	Si/Fe = 16	[8]
		Si/Fe = 11	[108]
		Si/Fe = 15–100	[109]
		Si/Fe = 38	[110]
4	Ti-FER	Si/Ti = 83–182	[111]
		Not mentioned	[113]
5	V-FER	Si/V = 120, 180	[112]
